# Advances in Genomics Approaches Shed Light on Crop Domestication

**DOI:** 10.3390/plants10081571

**Published:** 2021-07-30

**Authors:** Yang Zhao, Mengfan Feng, Dev Paudel, Tofazzal Islam, Aliya Momotaz, Ziliang Luo, Zifan Zhao, Ni Wei, Sicheng Li, Qing Xia, Bowen Kuang, Xiping Yang, Jianping Wang

**Affiliations:** 1 Guangxi Key Laboratory of Sugarcane Biology & National Demonstration Center for Experimental Plant Science Education, Guangxi University, Nanning 530005, China; zhaoyang@gxu.edu.cn (Y.Z.); mengfanfeng@st.gxu.edu.cn (M.F.); 1917301038@st.gxu.edu.cn (N.W.); lamina0130@126.com (S.L.); shandongxiaqing@163.com (Q.X.); kuangbowen97@163.com (B.K.); 2 State Key Laboratory for Conservation and Utilization of Subtropical Agro-Bioresources, Guangxi University, Nanning 530005, China; 3 Agronomy Department, University of Florida, Gainesville, FL 32610, USA; dev.paudel@ufl.edu (D.P.); luoziliang@ufl.edu (Z.L.); zifanzhao@ufl.edu (Z.Z.); 4 Institute of Biotechnology and Genetic Engineering (IBGE), Bangabandhu Sheikh Mujibur Rahman Agricultural University, Gazipur 1706, Bangladesh; tofazzalislam@bsmrau.edu.bd; 5 USDA-ARS Sugarcane Field Station, 12990 US Hwy. 441N, Canal Point, FL 33438, USA; aliya.momotaz@usda.gov

**Keywords:** crop, genomics approaches, domestication, application

## Abstract

Crop domestication occurred ~10,000–12,000 years ago when humans shifted from a hunter–gatherer to an agrarian society. Crops were domesticated by selecting the traits in wild plant species that were suitable for human use. Research is crucial to elucidate the mechanisms and processes involved in modern crop improvement and breeding. Recent advances in genomics have revolutionized our understanding of crop domestication. In this review, we summarized cutting-edge crop domestication research by presenting its (1) methodologies, (2) current status, (3) applications, and (4) perspectives. Advanced genomics approaches have clarified crop domestication processes and mechanisms, and supported crop improvement.

## 1. Introduction

Crops played a major role in human cultural evolution by causing a shift from a nomadic to a sedentary society. Hence, crops are suitable as evolutionary models illuminating genetic variation and selection. Crop domestication is a major agricultural advance ensuring food security for human society. Domestication is the result of phenotypic and genetic changes mediated by breeding. It involves multigenerational selection of plant traits favoring enhanced adaptation and acclimatization to farming management practices. Approximately 12,000 years ago, most economically important crops were domesticated [1,2]. Our ancestors instinctively selected crops that were easy to harvest and those with improved yield and flavor. These simple selection strategies helped pyramid important alleles and recombinants and resulted in naturally transformed plants with beneficial traits facilitating cultivation, breeding, storage, trade, and dissemination.

Of the ~5500 food crops worldwide, 15 contribute to ~70% of the total calories consumed by humans. Rice, wheat, and maize account for >50% of the calorie demand [3]. Up to 7000 known plant species are semi-cultivated or orphan crops [4]. These natural plant resources comprise a valuable pool of genetic material that could enable future crop breeding, increase food diversity, and respond to the new challenges of global climate change and population expansion [5]. The domestication of orphan and underutilized crop plants via recently developed biotechnologies such as genome-editing and genome-enabled approaches is highly promising in crop development for smart agriculture.

Most domesticated crop species share common traits such as increased yield and seed size and decreased dormancy and seed shattering. Though crop domestication is long and slow, only a few genes are involved in it, and some of them are conserved in various species [2,6,7,8,9,10]. Hence, both targeted re-domestication and *de novo* wild species domestication are feasible. In these processes, targeted genes are identified, introgressed, or modified to produce new cultivars. Unlocking the potential of wild crop species domestication will improve global food security and help realize certain sustainable development goals of the United Nations such as zero poverty (No. 1) and zero hunger (No. 2). Targeted domestication, crop improvement, and mass crop cultivation are generally cost-effective approaches towards these objectives. A concerted effort under the joint leadership of the Food and Agriculture Organization (FAO), Consultative Groups of International Agricultural Research Institutions, National Agricultural Research Institutions, and various governments is required for the research, popularization, and large-scale utilization of undomesticated crops with potential.

## 2. Genomic Methods for Studying Crop Domestication

To use crop breeding knowledge, technologies, and genetic resources effectively, it is necessary to understand the mechanisms of crop domestication. Several questions should be addressed to elucidate crop domestication. Where, when, and how did crop domestication occur? Did each crop go through single or multiple domestication processes? What was the genetic architecture of crop domestication? How did selection affect domesticated species? Some of these questions are being answered through genomic evidence.

### 2.1. Population Genomics

Population genetics and genomics have revealed that crops passed through four major stages during their evolution from wild progenitors to modern domesticated species [2]. These include (1) the onset of domestication when only one or a few wild progenitors with traits favored by humans were selected; (2) in situ propagation of selected wild progenitors to increase desirable alleles; (3) the spread and adaptation of cultivated populations to new environments; and (4) deliberate plant breeding to improve agronomic traits. During domestication, only a few individuals with traits serving human interests were selected from the wild progenitor population. Genetic drift caused by the founder effect and by selection reduced genetic diversity in domesticated crops. Genetic drift was assessed by comparing the genomes of domesticated crops and their wild relatives [11]. Advances in sequencing technologies and reduction of their costs have supported the publication of numerous high-quality studies on crop domestication using population genomics methods (Table 1). Huang et al. [12] compared the genome sequences of 446 wild and 1083 cultivated rice accessions. They found that *O. sativa japonica* originated in the middle of the Pearl River region in Southern China and was domesticated from a specific *O. rufipogon* population. There were 55 selective sweeps, and the genome signatures for selection during domestication were identified. They accounted for 5.1% of the genome regions (21.9 Mb) [12]. Hufford et al. [13] identified a few genes with strong selection in domesticated maize based on whole-genome resequencing of 75 wild, landrace, and improved maize lines. The authors also demonstrated that post-domestication diversity may have been recovered through introgression from wild relatives.

Advances in genetics, archeology, and their interdisciplinary areas have contributed to the clarification of crop domestication. Analyses of modern and ancient DNA have uncovered details about human and animal history. However, few studies have reported on the history of crop domestication, as there has been insufficient archeological evidence or DNA for genetic analysis [14]. Kistler et al. [15] sequenced 40 indigenous maize landraces and nine archeological samples from South America and compared them against 85 published maize genomes. The ancestral South American maize population was brought from its domestication center in Mexico before its domesticated traits were established. Multiple subsequent dispersal events led to maize diversity and biogeography. Scott et al. [16] prepared whole-genome sequences of a museum specimen of Egyptian emmer wheat chaff and demonstrated that ancient Egyptian emmers already shared a common origin with modern domesticated emmer even before the crop was introduced to Egypt. The foregoing results furnished evidence for early southeastern wheat dispersal and gene flow from wild to ancient Egyptian emmer.

Wang et al. [17] used a genome-wide variation map for 352 wild and domesticated cotton accessions. They scanned domestication sweeps covering 74 Mb of the ‘A’ subgenome and 104 Mb of the ‘D’ subgenome and found asymmetric subgenome domestication for directional selection of long fibers. Hufford et al. [13] conducted population genomic studies and discovered that 7.6% of all maize genomic regions were under selection during domestication. However, population genomics has certain drawbacks. First, genomic signals caused by domestication or improvement might be confused because genetic diversity is reduced in both cases. Second, certain crops have undergone multiple independent domestications wherein different genomic region layers may have been selected at different times. Hence, mixtures of samples from various domestication processes could obscure signals targeted for selection. Third, introgression may bilaterally occur between wild and domesticated crops and weaken signals identified through population genomics. Fourth, certain genomic signatures identified under domestication are not directly related to any agronomic traits, and their molecular mechanisms remain unclear. Hence, they must be validated by genome-wide association studies (GWAS), quantitative trait locus (QTL) mapping, map-based cloning, and functional targeted gene analyses.

### 2.2. Genome-Wide Association Studies (GWAS)

Genome-wide association studies (GWAS) or linkage disequilibrium (LD) mapping infer the genetic basis of domestication by identifying statistically significant associations between phenotypes and genotypes or between domesticated traits and sequence variants. GWAS explores natural diversity panels comprising unrelated individuals with historical LD [18].

There has been substantial progress in mapping the QTL underlying crop domestication. Huang et al. [12] performed a GWAS for leaf sheath color and tiller angle using 446 *Oryza rufipogon* accessions (Table 2). The strongest associations occurred near the known loci *OsC1* for coloration and *PROG1* for prostrate growth. They demonstrated that the mapping resolution was threefold higher for the wild rice population than for *O. sativa*, as the former had a relatively high LD decay rate. Wang et al. [17] conducted a GWAS on fiber quality-related cotton traits using 267 accessions with two million single nucleotide polymorphisms (SNPs) and a minor allele frequency > 0.05. They identified 19 association signals, of which 16 were new discoveries. Thus, the high-density SNP set was more powerful than the previous GWAS with simple-sequence-repeat markers. Forty-three association signals were identified for seven watermelon fruit quality traits [19]. There were 208 loci significantly associated with melon fruit mass, quality, and morphological traits [20].

Unlike population genomics, GWAS directly relates genomic regions to domesticated traits and facilitates the interpretation of domestication mechanisms at molecular level. GWAS usually has a higher targeted QTL mapping resolution than QTL mapping itself, as unrelated individuals can accumulate numerous genetic recombination events since their last divergence. Moreover, GWAS requires no genetic linkage maps, and the analysis is straightforward. However, GWAS depends on the target crop diversity panel, which is usually costly to collect and maintain. It is very difficult to develop a diversity panel with minimum population structure that is powerful enough for GWAS analysis. GWAS power may also be low when rare variants are causal mutations in a study panel [11].

### 2.3. QTL Mapping

Most evolutionarily important traits are quantitative. Phenotypic variation in these traits is the result of segregations at multiple QTL, the environment, and interactions between genes and the environment [21]. A QTL is a genomic locus correlating with phenotypic trait variation in a population and may be attributed to ≥2 genes on the same or different chromosomes. A QTL analysis provides the genetic basis for phenotypic variation, including gene locations, numbers, and magnitudes, and their mechanisms in a biparental segregating population [22]. QTL mapping has enabled successful identification and cloning of genes underlying domestication traits. It was the first and perhaps the most widely used method for localizing the genetic basis of a trait. Several QTL analyses revealed that wild and weedy genotypes were transformed into domesticated crop species. Pourkheirandish et al. [10] performed QTL mapping on three populations developed from crosses between domesticated barley and its wild progenitor and identified and cloned *Btr1* and *Btr2*, which control grain dispersal (Table 3). They demonstrated that 1-bp and 11-bp deletions in *Btr1* and *Btr2*, respectively, made the rachis non-brittle in domesticated barley. Doust et al. [9] analyzed shattering and flowering time in a foxtail millet mapping population and found that the alleles favored during domestication had larger phenotypic effects than the genetic background or the environment. Thus, recurrent selection in breeding can substantially increase domestication-related traits. Rice seed shattering QTLs were mapped on several chromosomes with a complex genetic architecture. *OsqSH1* was identified on chromosome 1 [23], and *SH4* was localized to chromosome 4 [24]. A later study supported that *qSH1* is epistatic to *SH4* in abscission process during seed shattering. In another study on molecular cloning, the non-shattering *SH4* allele was fixed in *O. sativa* ssp. *indica* and *O. sativa* ssp. *japonica* [25]. QTL analysis and map-based cloning showed *Sh1* on chromosome 1 encoded the YABBY transcription factor and underwent three independent mutations to form non-shattering domesticated sorghum [8]. QTL mapping is a straightforward and powerful approach to identify the genes controlling crop domestication.

### 2.4. Genome Editing Using CRISPR-Cas Technology

Clustered Regularly Interspaced Short Palindromic Repeats (CRISPR) is used in gene manipulation and is revolutionary in biological research [26,27]. CRISPR was first discovered in 1987 and recognized as an adaptive immune system in archaea and bacteria [28,29]. In 2012, several research groups independently discovered that CRISPR and its associated protein (Cas) constitute a powerful genome-editing technology inducing precise DNA breaks at targeted genome locations in any living cell [26,27]. The CRISPR-Cas tool was first successfully used for plant genome editing by three independent groups in 2013 [30,31,32]. CRISPR-Cas has been used for programmable gene/epigenome editing and transcriptome regulation in plants [33,34,35]. It can also edit multiple genes (multiplexing) through simultaneous multiple guide RNA (gRNA) delivery and expression [30]. Through multiplex gene editing, CRISPR promotes basic research, accelerates plant breeding, and facilitates plant domestication and germplasm development. CRISPR-Cas can fine-tune and knock out master switches in undomesticated wild crops, enhance genomic diversity, and facilitate *de novo* domestication in one generation or a few generations [33].

The power of CRISPR-Cas toolkit in *de novo* plant domestication was demonstrated in several studies [36,37,38,39]. Zsögön et al. [39] used CRISPR-Cas to show genome editing for several domesticated genes in tomato such as *SP*, *SP5G*, *SlCLV3*, and *SlWUS* (Table 4). Furthermore, CRISPR was extended to the shuffling chromosome and used to stack multiple alleles into one tightly linked locus. CRISPR-Cas9-mediated induction of heritable chromosomal translocation was demonstrated in *Arabidopsis* [40]. Li et al. [38] applied CRISPR-Cas multiplex genome editing to four *Solanum pimpinellifolium* accessions that were salt-tolerant or highly resistant to bacterial spot disease. The genome-edited plants acquired targeted domestication traits while retaining their abiotic and biotic stress tolerance. Therefore, CRISPR-Cas multiplex editing introduces novel and retains existing plant traits and develops an ideal crop [41]. CRISPR-Cas technology could provide precise and customized modifications conducive to plant breeding. Successful application of CRISPR-Cas in tomato and wild cherry indicated that this technology could domesticate in one generation new crops resilient to environmental change [37,42,43].

The benefits of *de novo* orphan crop domestication via CRISPR-Cas gene editing include (i) high precision and accuracy, (ii) short variety development time, (iii) transgene-free product, (iv) high crop yield and nutritional value, and (v) crop resistance to biotic and abiotic stresses. However, the major challenges of *de novo* domestication via genome editing include (i) limited genomic information for wild relatives, (ii) lack of a transformation system for wild relatives, (iii) fitness cost, and (iv) limited public acceptance of genetically modified organisms (GMO).

## 3. Current Status of Research on Crop Domestication

### 3.1. Domestication Centers and Their Spread

**Figure 1:**
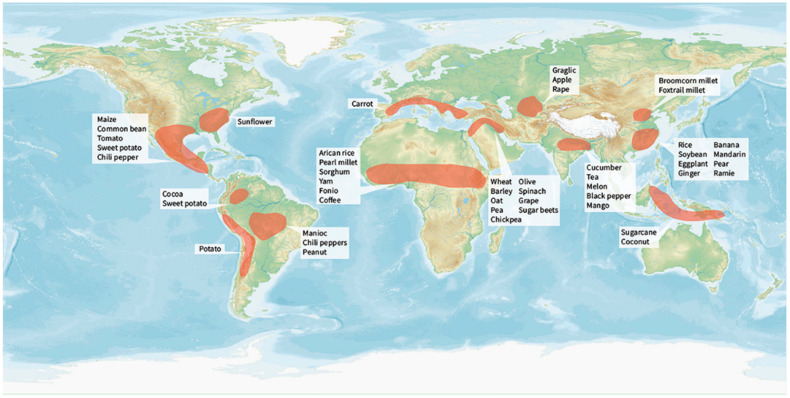
Global food crop origin and domestication. Shaded regions indicate approximate locations of centers of crop origin and domestication.

Around 12,000 years ago, human-guided crop domestication occurred independently in the Middle East, the Fertile Crescent, China, Mesoamerica, the Andes, Near Oceania, Sub-Saharan Africa, and Eastern North America [2,47]. Of the 2500 domesticated plant species distributed in 160 families, 250 are fully domesticated [2]. Table S1 and Figure 1 show the origins and major cultivation zones of global domesticated food crops. Some of them are widely spread across several regions, whereas others are more regionally or locally important. The domestication, spread, and cultivation of food crops have demonstrated the transition from hunter–gatherer to agrarian societies.

Maize is one of the most important food crops worldwide, and extensive research progress has been made on its domestication. Starch grain and phytolith evidence indicated that maize was first domesticated from wild Balsas Teosinte (*Zea mays* ssp. *parviglumis*) in Mexico ~9000 years ago [48]. After partial domestication in Mexico, maize traversed Panama, arrived in Central America ~7500 years ago, and was brought to South America ~6500 years ago. In South America, maize was fully domesticated at several independent locations ~6500–4000 years ago [15]. Two major maize movements from Mesoamerica to South America were deduced, as the Pan-American lineage shared excess ancestry with *parviglumis* compared with the strictly South American lineage. Maize was brought to the United States ~4000 years ago. In the 15th century, European colonies spread maize through the Americas and, thence, globally.

The spread of domesticated crops is slow and complex. It is affected by shifts in human society, farming practice improvements, and plant adaptability to various climates. Plants adapted to different global climates in an east to west trajectory. Archeological evidence supports that maize spread from west to east across the Amazon, which was a secondary improvement center for partially domesticated maize [15]. However, since its initial domestication in Mexico maize has spread across the Americas, including the south-to-north and lowland-to-highland directions. Gene flow from the wild relatives of maize might have improved its adaptation to various ecological niches [49].

### 3.2. Domestication Theory

The study of the inheritance of domestication genes raises a crucial question, namely, does selection act on existing variations segregating in ancestral wild populations or *de novo* mutations? Current research supports the possibility that selection acts on both variations and mutations. However, numerous domesticated traits arise from existing variations in ancestral wild populations [2]. Standing variations apparently allow rapid evolution of populations, as they lack the lag periods characteristic of *de novo* mutations [50]. Alleles selected from standing variations occur at low to moderate frequency in wild progenitors, and there are weak signatures in the genome for the selection of old mutations. Therefore, determining which mutations are affected by selection in the domestication process may help clarify the nature of selective sweeps and the rate of crop evolution.

Traits selected in domestication may distinguish crops from wild progenitors. This mechanism is known as the domestication syndrome [51]. Different crops and the same crop with multiple origins shared the same domesticated phenotypes such as loss of seed dormancy and non-shattering seeds. The existence of convergent phenotypes raises the questions as to how selection behaves in domestication and whether the same or different genes are affected by it. Molecular parallelism might explain this phenomenon. Multiple mutations in the same or different genes resulting in the same phenotype have been independently selected for domestication. Three non-shattering haplotypes at *SH1* locus were characterized in domesticated sorghum and were distributed among sorghum landraces. Thus, multiple domestications of a species may occur [8]. *SH1* was under selection for rice and maize domestication as well. However, the domestication phenotype can also be controlled by different genes. Doust et al. [52] identified novel genes controlling branching in foxtail millet. In contrast, the *teosinte brached1* ortholog had only a minor effect on this trait. Lai et al. [53] examined genome resequencing data from wild and domesticated maize and sorghum accessions and showed that the number of candidate domestication genes with parallel selection signatures was not significantly higher than that expected by chance. Certain major genes with large effects might have been repeatedly targeted by domestication selection. Alternate genes may have also produced similar phenotypes in different crop species.

Four demographic crop domestication models were proposed to elucidate the domestication process [2]. In an earlier model, a single domestication event resulted from strong selection in a small wild progenitor population and caused total reproductive isolation between the wild and domesticated species. However, archeological and genetic data suggested that genetic bottlenecks vary among crop species and introgression occurs between crops and wild relatives. Therefore, the model was modified to alternate versions wherein a single domestication event occurred with gene flow between crops, or multiple domestication events with gene flow occurred, or domestication events from interspecific hybridization occurred and were followed by clonal propagation. Crops assessed by these alternate models were reviewed by Meyer and Purugganan [2].

### 3.3. Genetic Architecture and the Molecular Basis of Genes Mediating Crop Domestication

Several QTL or GWAS studies identified genes underlying the domestication syndrome in various crops (Table S2), of which 40.5% were regulatory (transcription factors or co-regulators) and 56.0% were structural (enzymes or other proteins). Certain genes such as *Prog1* and *Prog7* control domestication traits such as prostrate rice tillers. However, the advancement of population genomics disclosed that large genomic regions such as 7.6% of the maize genome [13] and 6.9% of the cotton genome [17] were under domestication selection. This paradox could be explained by the inherent limitations of QTL or GWAS. A large proportion of the missing heritability cannot be explained by small populations containing only a few recombinants with low enough marker density to capture significant genomic regions.

Meyer and Purugganan [2] proposed that a domestication gene has a clear function associated with a domesticated trait, is under positive selection, and is fixed or almost entirely fixed at the causative mutation in all lineages under a single domestication event. The authors compiled 60 genes involved in domestication or diversification, of which 40 (66.7%) encoded transcription factors or co-regulators, 14 (23.3%) encoded enzymes, and 6 (10%) encoded transporter proteins and ubiquitin ligase. These genes can encode various traits of which some are involved in the domestication of different crops. The primary effects of causative mutations in the aforementioned genes include the creation of nonsense mutations, premature truncations, other mutations resulting in null function, *cis*-regulatory mutations, and missense mutations. Mutations with large phenotypic effects are the most common functional changes.

## 4. Domestication in Modern Crop Breeding

### 4.1. Rice

Rice is one of the most important food crops in the world, and it is also a model system to study crop domestication. Though there are tons of literature discussing rice origin and domestication, the origin and history of rice domestication remain controversial. Despite this, it is widely accepted that selections together with introgression shaped the genomes of cultivated rice [54]. With the achievements of researches on rice domestication, their applications in modern rice breeding are impressive. To meet the growth needs for food under the global climatic challenges, breeders combine genetic resources of domestication genes with those containing multiple valuable alleles to create superior cultivars [55]. In traditional crosses of diverged cultivars or germplasm, the process of selection of robust agronomic traits and removing unfavorable backgrounds could be accelerated by using molecular markers developed according to domestication genes.

Genome editing technology, which can efficiently modify target genomes predictably and precisely, is no doubt a revolutionary tool to perform molecular domestication to obtain desirable traits in laboratory [56,57]. Using this technology in rice, scientists successfully reduced seed shattering by editing *qSH1* gene [58], broke down seed dormancy by knockout *OsVP1* [59], and developed superior alleles of yield genes by editing *Gn1a* and *DEP1* genes [60]. These studies have proven the potential to improve target traits substantially in rice by editing single or a few domestication genes. Moreover, cis-regulatory elements are alternative targets for editing, which can tune gene expression levels, timing, and tissue specificity, but avoids any detrimental pleiotropic effects due to mutations in coding regions [61]. Recently, a strategy to *de novo* domesticate wild allotetraploid rice was described, and six agronomical traits were improved rapidly by genome editing of target genes [62], demonstrating the possibility to develop this polyploid wild rice to a food crop. Though still at the beginning stage, *de novo* domestication based on advanced genomics approaches shortens the process of domestication to a few years, which opens up a gate to utilize wide genetic resources in a precise way.

### 4.2. Tomato

Tomato (*Solanum lycopersicum*) originated in the Andean region, and its domestication occurred before the 15th century. Intense tomato domestication occurred in Europe in the 18th and 19th centuries [63], and tomato cultivar improvement has been ongoing since then. Wild tomato has large genetic diversity and has been extensively studied to characterize certain traits favorable for breeding [64,65]. In contrast, cultivated tomato has very low genetic diversity and has <5% of the genetic variation in their wild relatives [66]. The domestication syndrome has been studied for this crop, and several QTLs underlying growth habit and fruit size were identified [67,68,69].

Advances in genome editing and crop domestication enable plant geneticists to target certain genomic sites in wild plants and rapidly create improved cultivated crops. Zsögön et al. [39] edited six loci in wild tomato (*Solanum pimpinellifolium*) and generated highly productive progeny that could serve to breed improved cultivars. The six loci that were previously considered vital to tomato domestication regulated general plant growth habit (*SELF-PRUNING*) [70], fruit number (*MULTIFLORA*) [71], fruit shape (*OVATE*) [72], fruit size (*FASCIATED* and *FRUIT WEIGHT 2.2*) [73,74], and nutritional quality (*LYCOPENE BETA CYCLASE*) [75]. These genes were targeted by multiplex CRISPR-Cas9, and loss-of-function alleles were generated [39]. The T_1_ lines were successfully edited for *SELF-PRUNING* (*SP*), *OVATE* (*O*), *FRUIT WEIGHT 2.2* (*FW2.2*), and *LYCOPENE BETA CYCLASE* (*CycB*). Compared with the wild type, the engineered plants had higher fruit numbers per truss, enhanced yield, a fourfold increase in fruit locule number, a 200% increase in fruit weight, 100% higher lycopene content, and stable β-carotene and lutein content [39]. Moreover, Brix value, fruit shape, and locule number were uniformly inherited in T_2_ and T_3_. Hence, the engineered traits were stable, and the wild tomato was successfully domesticated [39]. CRISPR-Cas9 was also effectively used to mutate tomato domestication gene orthologs controlling plant architecture, flower production, and fruit size in ground cherry (*Physalis pruinose*) [37], a solanaceous orphan crop.

### 4.3. Potato

Potato (*Solanum tuberosum*) is one of the most important food crops worldwide. However, cultivated potato varieties are autotetraploid and vegetatively propagated. Consequently, breeding efforts for tuber yield and quality improvement are very limited. Most potato germplasms bearing alleles controlling agronomically important traits are diploids [76]. The reinvention of inbred diploid varieties has been proposed to overcome this limitation and accelerate breeding [77]. Most diploid potato species are gametophytically self-incompatible. This trait is controlled by S-RNase genes. Recent attempts have been made to edit S-RNase genes and achieve self-compatible diploid potato varieties [78,79]. The potato genome resource and diploid potato line sequencing data identified S-RNase orthologs. CRISPR-Cas9 guided S-RNase gene knockout and successfully created self-compatible diploid potato lines that could be pollinated and generate enough seed for propagation. Thus, the domestication of wild diploid potato into an inbred crop is a novel strategy in potato genetic improvement.

### 4.4. Orphan Crops

Orphan crops are semi-cultivated species with limited regional importance such as dry bean (*Vigna* spp.) and lupin. They are often relatively less productive, not optimized for modern agriculture, and infrequently studied by the research community [80]. However, unlike several major cultivated crops, they have wide biodiversity and are adapted to poorly controlled or harsh environments. Orphan crops provide nutritional benefits and may tolerate extreme heat or cold [81]. In view of constant pressure from climate change and increased demands for food by growing populations, orphan crop domestication may become vital to food security in the future.

Ground cherry (*Physalis pruinose;* Solanaceae) is indigenous to Mexico and South America and an orphan crop distantly related to tomato [82]. However, its small fruits fall to the ground because of stem abscission, and the plant has a sprawling growth habit. Hence, its productivity is limited. Lemmon et al. [37] developed an *Agrobacterium tumefaciens*-mediated transformation system to enable editing of the domestication genes related to the aforementioned traits of ground cherry. The genomic resources were enriched by whole-genome and RNA sequencing. Orthologs of the tomato florigen repressor genes *SELFPRUNING* (*SP*) and *SELF-PRUNING 5G* (*SP5G*) in ground cherry (*Ppr-SP* and *Ppr-SP5G*) were selected for knockout in the CRISPR-Cas9 experiment. In tomato, mutations in these genes produce a compact plant architecture and flower and fruit burst. The *Ppr-SP* knockout plants were extremely compact and presented with a slight relative increase in fruit production. The *PprSP5G* knockout plants displayed a compact structure and significantly enhanced fruit production because of moderate sympodial shoot termination. The *CLAVATA* (*CLV*) ortholog (*Ppr-CLV3*) modifies fruit size by domesticating the locule number, and it was targeted with CRISPR-Cas9. The *Ppr-CLV3* mutants exhibited a 24% relative gain in fruit mass growth. In the future, other important domestication genes such as *JOINTLESS-2* (fruit abscission) will also be edited to improve the agronomic traits of ground cherry.

## 5. Crop Domestication Perspectives

From the first plant domestication at least 12,000 years ago to the present day, numerous crops have been subjected to human selection especially for the purpose of continuously increasing yield. However, domestication and modern plant breeding have steadily reduced genetic variation in crops. Consequently, modern cultivars are highly susceptible to biotic and abiotic stresses such as drought, heat, insects, and disease [83]. Wild germplasms of cultivated species have wide genetic variation and stress tolerance traits that should be exploited in modern breeding programs to develop resilient cultivars.

Domestication may involve increasing the size of certain organs such as the fruits and seeds. Therefore, genes regulating cell division, meristem size, and patterning are vital [84]. Other morphological traits such as flower number, flowering time, and nutrient composition are also important traits for selection. There is a wide range of gene functions under selection. Certain genes might have been under selection for traits never considered as targets during crop domestication. As a rule, only a few genes play major roles in domestication. Nevertheless, our understanding of domestication is constantly being reshaped by new discoveries. In the coming decade, population genomics, GWAS, and genome-editing tools will clarify genomic signatures in domestication and greatly enhance our ability to domesticate wild plant species. For example, candidate domestication genes in wild and domesticated species may be sequenced to identify selective population sweeps and functionally associate them with SNPs via GWAS. Affordable sequencing technology will enable fine mapping in orphan crops and accelerate their breeding. Regions of high divergence between cultivated and wild species may also be identified so they can be associated with domestication. There remains much to be learned about how domestication changes crop genome composition.

An important objective going forward is to determine whether this new domestication knowledge can serve as guidance for future plant breeding efforts. Current advances in plant breeding appear to indicate that we are heading in the right direction. Genome editing-tools have advanced our understanding of domestication genes and enabled us to develop new cultivars by directly incorporating domestication-related genes. For instance, the engineered ancestral progenitor of wild tomato differed in terms of fruit morphology, size, number, and nutritional value from the widely cultivated tomato [39]. Efficient crop *de novo* domestication will depend on the availability of characterized domestication genes, effective transformation methods, and open access to genome-editing technologies. And more importantly, successful *de novo* domestication should integrate genetic tools with agronomic and cultural drivers to accommodate the newly designed crops to adapt to dynamic environments and agronomic practices, and to be accepted by consumers [85].

From the aspect of genetics, rapid domestication may be realized via comparative genomics of various crop accessions mediated by next-generation sequencing and the inherent synteny between crops. This process was applied to ground cherry using tomato gene orthologs [37]. The exclusive application of domestication-related genes has helped domesticate orphan crops and develop new varieties of cultivated crops. Allele mining and gene or QTL cloning can recover superior alleles that do not pass through domestication bottlenecks [86]. Plant breeding has benefitted from the recommendation of Vavilov to collect and maintain wild crop relatives in gene banks [87]. Knowledge acquired from omics technology also complements traditional plant breeding approaches. Plant breeders with access to large datasets can develop new cultivars in a ‘breeding by design’ process using CRISPR-Cas genome editing [88,89,90]. Using the various tools available to them, breeders can enhance crop productivity, nutritive value, and biotic and abiotic stress tolerance.

## Figures and Tables

**Table 1:** The application of population genomics to crop domestication.

Crop	Population Type	Population Size	Key Statistic	Discovery	Ref.
Rice	Ancestral progenitor; cultivated indica and japonica varieties	1529	Sequence diversity (π) population-differentiation (F_ST_), cross-population extended haplotype homozygosity (XP-EHH)	Identify 55 domestication sweeps, and reveal the domesticaiton and development of cultivated rice	[12]
Maize	Wild, landraces and improved maize lines	75	π, ρ, F_ST_, Tajima’s D, normalized Fay and Wu’s H, and a composite likelihood approach (XP-CLR)	Evidence of recovery of diversity after domestication, and stronger selection for domestication than improvement	[13]
Maize	Ancient samples, modern maizes landraces, and teosintes	134	Mutation load, *D*-statistics, and f3 and f4 statistic	Reveal domestication center and human-mediated spread of maize	[15]
Wheat	Ancient and modern domestic emmer	64	Haplotype structure	Uncover the history and diversity of emmar wheat	[16]
Cotton	Wild and domesticated cotton accessions	352	π, F_ST_, and XP-CLR	Identify 93 domestication sweeps	[17]

**Table 2:** The application of GWAS to crop domestication.

Crop	Domestication Trait	Population Type	Population Size	Genotype Method	Model	Discovery	Ref.
Rice	Leaf sheath color and tiller angle	Ancestral progenitor *Oryza rufipogon*	446	Whole genome resequencing	Compressed mixed linear model	Identify assoicaitons for *OSC1* and *PROG1*	[12]
Cotton	Fiber quality related traits	Cotton accessions	267	Whole genome resequencing	Compressed mixed linear model	Identify 19 assoicaiton signals (16 were new)	[17]
Watermelon	Fruit quality traits	Cultivated and wild watermelon accessions	414	Whole genome resequencing	Linear mixed model algorithm	Identify 43 associaiton signals (35 were new)	[19]

**Table 3:** The application of QTL to crop domestication.

Crop	Domestication Trait	Population Type	Population Size	Marker	Discovery	Refs.
Rice	Seed shattering	F_2_	304	RFLP, RAPD, SNP, SSR	Localized the gene *qSH1* and gene *sh4*	[23,24]
Barley	Rachis non-brittle	F_2_	>10,000	SNP	Localized the gene *btr1* and *btr2*	[10]
Foxtail millet	Shattering and flowering time	Recombinantinbred line	182	SNP, SSR, and sequence-tagged site markers	Two significant QTLs	[9]

Note: SSR, simple sequence repeat; RFLP, restriction fragment length polymorphisms; RAPD, random amplified polymorphic DNA.

**Table 4:** The application of CRISPR-Cas to crop domestication.

Crop	Domestication Traits	Target Gene	Method	Discovery	Ref.
Rice	Panicle length, grain size, cold tolerance	*OsPIN5b*, *GS3*, *OsMYB30*	CRISPR-Cas9 system edits three genes simultaneously	Higher yield and better cold tolerance in gene-edited rice	[44]
Wheat	Grein length, weight and yield, TKW, Inflorescence architecture, branching and tillering	*Tagasr7-A1 (-B1* and *–D1), TaDEP1, TaNAC2, TaPIN 1,* and *TaLOX2*	Transient expression of CRISPR-Cas9 in callus cells	Changes on target traits in wheat callus and regeneration of plants	[45]
Tomato	Fruit size, number and nutrition	*SP* *, O, FW2.2* *, CycB*	CRISPR-Cas9 system edits six genes simultaneously	Gene-edited tomato has at least a threefold increase in target traits	[39]
Cucumber	Carpel development	*CsWip1*	Optimized CRISPR/Cas9 system with *CsU6* promoter and GFP	Seven times more female flowers in gene-edited cumcumber	[46]

Note: GFP, green fluorescent protein; TKW, thousand kernel weight.

## Data Availability

Not applicable.

## Supplementary Materials

The following are available online at https://www.mdpi.com/article/10.3390/plants10081571/s1, Table S1: Global domesticated food crops, their origins, and major cultivation zones, Table S2: Food crop domestication genes.
